# A new species of *Eutettix* (Hemiptera, Cicadellidae, Deltocephalinae) from Wisconsin

**DOI:** 10.3897/zookeys.557.5939

**Published:** 2016-01-28

**Authors:** Stuart H. McKamey

**Affiliations:** 1 USDA Agricultural Research Service, Systematic Entomology Laboratory, c/o NMNH, MRC-168, Smithsonian Institution, P.O. Box 37012, Washington, DC, 20013-7012, U.S.A.

**Keywords:** Leafhopper, new species, Athysanini, Nearctic, Wisconsin, Quercus

## Abstract

*Eutettix latoides* **sp. n.**, is described from central Wisconsin. It most closely resembles the Californian species *Eutettix latus* Hepner, and was collected from *Quercus ellipsoidalis*.

## Introduction

The genus *Eutettix* Van Duzee pertains to the deltocephaline tribe Athysanini, the largest tribe of the family, which defies easy diagnosis. The tribe generally consists of a diverse assortment of 228 genera with Y-shaped connectives in the male genitalia that lack characteristics that would place them in other tribes (Zahniser and Dietrich 2013).

As with many athysanine genera, *Eutettix* itself is more readily distinguished than its tribe. They are robust, somewhat flattened leafhoppers with the head in dorsal view slightly broader than the pronotum, with a faint transverse furrow just behind the rounded anterior margin, and the anterior margin with several to many transverse striations or irregular carinae. The ocelli are located on the anterior margin close to the eyes. The pronotum has very short lateral margins and bears transverse striae. The forewing has the appendix restricted to the anal vein area, with flat (non-carinate) veins, and three anteapical cells. The abdominal tergum X is sclerotized. The male pygofer is setose with a large single or double hooklike spine within it. The subgenital plates are triangular with uniseriate macrosetae.

Although 127 species were originally described in *Eutettix*, it is currently considered a coast-to-coast Nearctic genus containing 56 valid species and subspecies, 31 of which are endemic to the United States, 15 endemic to Mexico, and three species are shared between the two countries. There are seven species left over from the out-dated, wider interpretation of the genus that are still waiting for proper generic placement: *Eutettix botelensis* Matsumura from Taiwan; *Eutettix elongatus* Melichar from the Republic of the Congo; *Eutettix fulminans* Melichar from Indonesia; *Eutettix marquezi* Merino from the Philippines; *Eutettix mimicus* Osborn from Bolivia; *Eutettix quadripunctatus* Melichar from Somalia; and *Eutettix ramosus* Melichar from Tanzania.

Hepner (1942) provided the first and only revision of the species of the United States, describing 18 new species, three new subspecies, three new synonyms, and descriptions and genitalic photographs of all species. He also listed five species that did not belong to *Eutettix* but retained them in the genus for convenience until their generic affinities were better understood – all have since been reassigned to other genera, including one to the genus *Ollarianus* Ball, under which one current *Eutettix* species (*Eutettix rubianus* [Ball]) was originally described. Sixteen Mexican species were added by DeLong and Harlan (1968) and DeLong (1980). No subsequent species have been described from anywhere in the last 33 years. In the current work a new species of *Eutettix* is described, bringing the total number of Nearctic species to 50.

## Methods

Multiple images per view were captured using a Microvision System with an AT-200GE videocamera mounted on a Leica 10447176 Planapo 1.0x/WO 97mm lens, and compiled using Cartograph 8.0.6 software. The resulting images were cleaned using Adobe Photoshop CS 3 version 10.0.1.

Terminology follows Zahniser and Dietrich (2013), also available online in Zahniser’s interactive key to deltocephaline tribes (http://imperialis.inhs.illinois.edu/zahniser/JZKeys.asp).

The holotype is deposited in the United States National Museum of Natural History in Washington, DC (USNM).

## Results

### Eutettix latoides sp. n.

Taxon classificationAnimaliaHemipteraCicadellidae

http://zoobank.org/45B41234-5AEC-411A-AD44-398D5C5CD0D7

#### Diagnosis.

male with internal pygofer hook bifurcate distally, its ventral branch approximately five times wider than its dorsal branch.

#### Measurements.

Length of male with forewings in repose 4.4 mm, maximum width of pronotum 1.4 mm.

#### Description.

**Figures 1–7.**
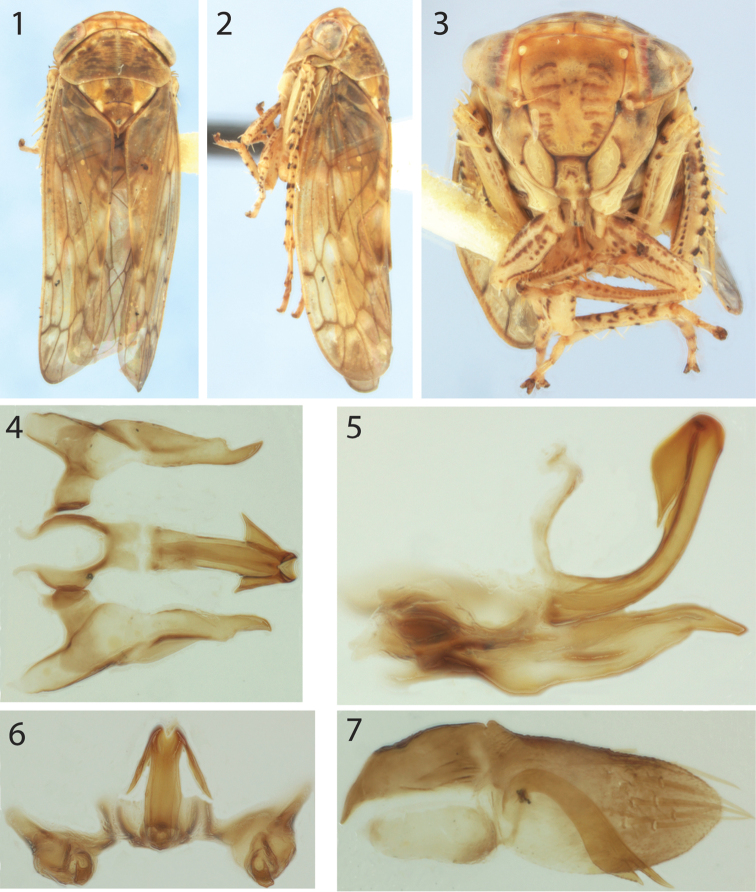
*Eutettix latoides* sp. n., holotype. **1–3** habitus from dorsal, lateral, and anterior views, respectively **4–6** aedeagus, styles, and connective from dorsal, lateral, and posterrior views, respectively **7** Right side of pygofer, internal view, showing its diagnostic pygofer hook (left side removed).

***Head.*** Slightly wider than pronotum, anterior margin rounded in lateral view, with transverse striations between ocelli in anterior view, and, in dorsal view (Fig. 1) parallel margined, vertex with shallow furrow just behind anterior margin; frontoclypeal suture distinct (Fig. 3), clypeus slightly wider distally, frontoclypeus not tumid (Fig. 2); ocelli on anterior margin of head close to eyes, separated from them by nearly their width (Fig. 3). Thorax. Pronotum transversely striate, posterior margin weakly concave, lateral margin shorter than basal width of eye (Fig. 1); forewing macropterous with veins not raised, distinct appendix limited to anal margin, three anteapical cells, 2^nd^ slightly constricted medially, venation not reticulate distally, A1-A2 crossvein absent; prothoracic femur (Fig. 3) with many small setae in row AV and intercalary row, AM1 seta present, dorsal surface rounded, not sharply carinate along AD and PD margins; metathoracic femoral apex macrosetal formula 2+2+1.

***Male terminalia.*** Pygofer in lateral view bluntly pointed; pygofer hook (Fig. 7) arising basoventrally within pygofer, curving gradually dorsoposteriorly then abruptly ventroposteriorly, bifurcate apically, ventral branch finely serrate and gradually acuminate, approximately 5× wider than dorsal branch, which has subparallel sides until acute apex. Aedeagus (Figs 4–6) in lateral view narrow throughout, lacking basal or median processes, bearing a pair of anterior apical leaflike processes slightly longer than half of aedeagal shaft, directed ventrolaterally, gonopore apical. Style with small basal lobe, pointed apex directed posteriorly. Connective basally broad, articulated with aedeagus, in dorsal view Y-shaped.

***Color.*** Irregularly fuscous throughout, darker along transverse furrow on head frontoclypeal suture and vertex, on forewing cubitus apex and both r-c crossveins, and on legs setal bases.

***Female.*** Unknown.

#### Distribution.

United States: central Wisconsin.

#### Probable host.

*Quercus ellipsoidalis* E.J. Hill (Northern pin oak or Hill’s oak). Because the label does not indicate how the specimen was collected from the oak, its host must be considered tentative until further specimens are collected. Nevertheless, it may indeed feed on oak. Hepner (1942) reported three other *Eutettix* species that have been collected from oaks: *Eutettix querci* Gillette and Baker, from *Quercus undulata* Torr. (Wavyleaf oak), and *Eutettix querci albus* Hepner and *Eutettix prinoides* Hepner, both from *Quercus prinoides* Willd. (Dwarf Chinkapin oak).

Holotype (USNM): male, with labels “[Wisconsin Rapids,] Wood Co., Wis. / Griffith St[ate]. Nursery / VII-22-1948 / R.D. Shenefelt Ray”, “coll. from / Quercus ellipsoidalis / normal”, and “HOLOTYPE / Eutettix / latoides / S.H. McKamey.” Brackets indicate inferred data not on labels. Georeference: 44.3408°N; -89.7349°W (DD).

#### Etymology.

The name is a combination of “*latus*” and the Greek suffix “-*oides*,” in reference to the resemblance of the new species to *Eutettix latus*, as discussed below.

## Discussion

In Hepner’s (1942) key to species, the new species would key to couplet 5 because it is less than 4.75 mm, but has features inconsistent with either portion of the couplet: the dorsal branch of the pygofer hook is not falcate, as in *Eutettix rugosus* Hepner, but the branches are subequal in length and the ventral branch is not curved anteriorly, as in *Eutettix subspinosus* Hepner. The internal pygofer hook most resembles that of *Eutettix latus* Hepner (USNM paratypes examined), but in that species the pygofer hook bifurcates just after its ventral curve, with both branches narrowing to equally acute apices. Another feature distinguishing *Eutettix latoides* from *Eutettix latus* is that the latter species has the aedeagal shaft broad with a large medial anterior projection, and its apical pair of processes are approximately one third the size of those in *Eutettix latoides*.

## Supplementary Material

XML Treatment for Eutettix latoides
